# *trans*-Di­chlorido­tetra­kis­(pyridine-4-carbo­nitrile-κ*N*)ruthenium(II) toluene hemisolvate

**DOI:** 10.1107/S2414314626009375

**Published:** 2026-09-25

**Authors:** Frank S. Goodavish, Sebastião A. Martin, Eric W. Reinheimer, Bradley W. Smucker

**Affiliations:** a Austin College, 900 N Grand Avenue Sherman TX 75090 USA; b Rigaku Oxford Diffraction, 9009 New Trails Dr., The Woodlands, TX 77381, USA; Benemérita Universidad Autónoma de Puebla, México

**Keywords:** crystal structure, ruthenium(II), building block, 4-cyano­pyridine, voids

## Abstract

The title compound, [RuCl_2_(C_6_H_4_N_2_)_4_]·0.5C_7_H_8_, resulted from the reaction of four equivalents of pcp (pyridine-4-carbonitrile) with ruthenium(II) chloride to form a propeller-like arrangement, which is positioned along the twofold axis of the *C*2/*c* space group. The packing of the complex in the structure, resulting from the overlap at the carbonitrile groups of the complexes, results in a grid-like assembly with Ru⋯Ru diagonal distances of 17.0973 (9) and 20.0289 (5) Å, and a near 50% calculated void volume.

## Abstract 2

The propeller-like title complex packs with overlapping carbonitrile groups to form a grid with a significant void volume.

## Graphical abstract

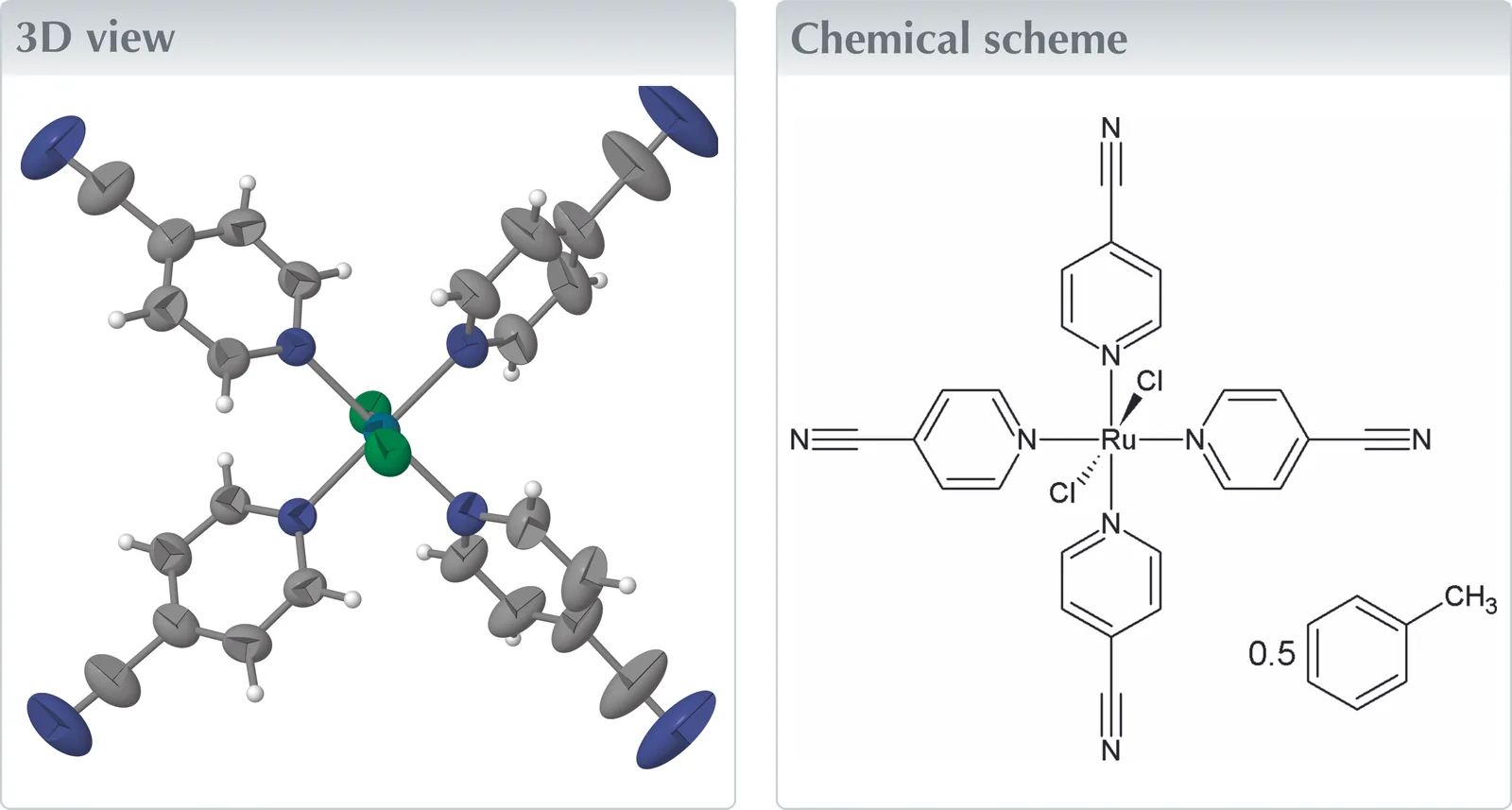

## Structure description

Ruthenium(II) building blocks consisting of four pyridyl-type ligands, such as [Ru(*N*-methyl-4,4′-bipyridinium)_4_Cl_2_]^4+^ (Cadranel & Hodak, 2015 ▸), have been utilized to engineer coordination networks including tetra­substituted pyrazine (Carlucci *et al.*, 2002 ▸) or 4,4′-bi­pyridine, which was used to explore new electrochemical devices (Vertova *et al.*, 2007 ▸). In a course-based undergraduate research experiences (CURE), similar to a recently reported program incorporating X-ray crystallography in education (Abrahams *et al.*, 2023 ▸), we have been investigating the syntheses and structural characterization of these types of neutral ruthenium(II) chloride building blocks with tetra­kis­(pyrazine) (Nesterov *et al.*, 2012 ▸), tetra­kis­(4-meth­oxy­pyridine) (Reinheimer *et al.*, 2023 ▸), and the disubstituted 4-di­methyl­amino­pyridine and dimethyl sulfoxide (Park *et al.*, 2024 ▸) ligands. We report herein the tetra­substituted coordination of pcp (pyridine-4-carbo­nitrile (Enkelmann *et al.*, 2021 ▸) with ruthenium(II) chloride. The coordination of a single pcp ligand with Ru^II^ has been utilized to investigate DNA binding (Singh *et al.*, 2007 ▸) and organometallic cancer cell cytotoxicity (Wang *et al.*, 2005 ▸). Other reported propeller-like structures of *trans*-[*M*(pcp)_4_(NCS)_2_] include *M* = Mn^II^ (Wellm *et al.*, 2020 ▸) and *M* = Ni^II^ (Clegg & Harrington, 2016 ▸).

**Figure 1:**
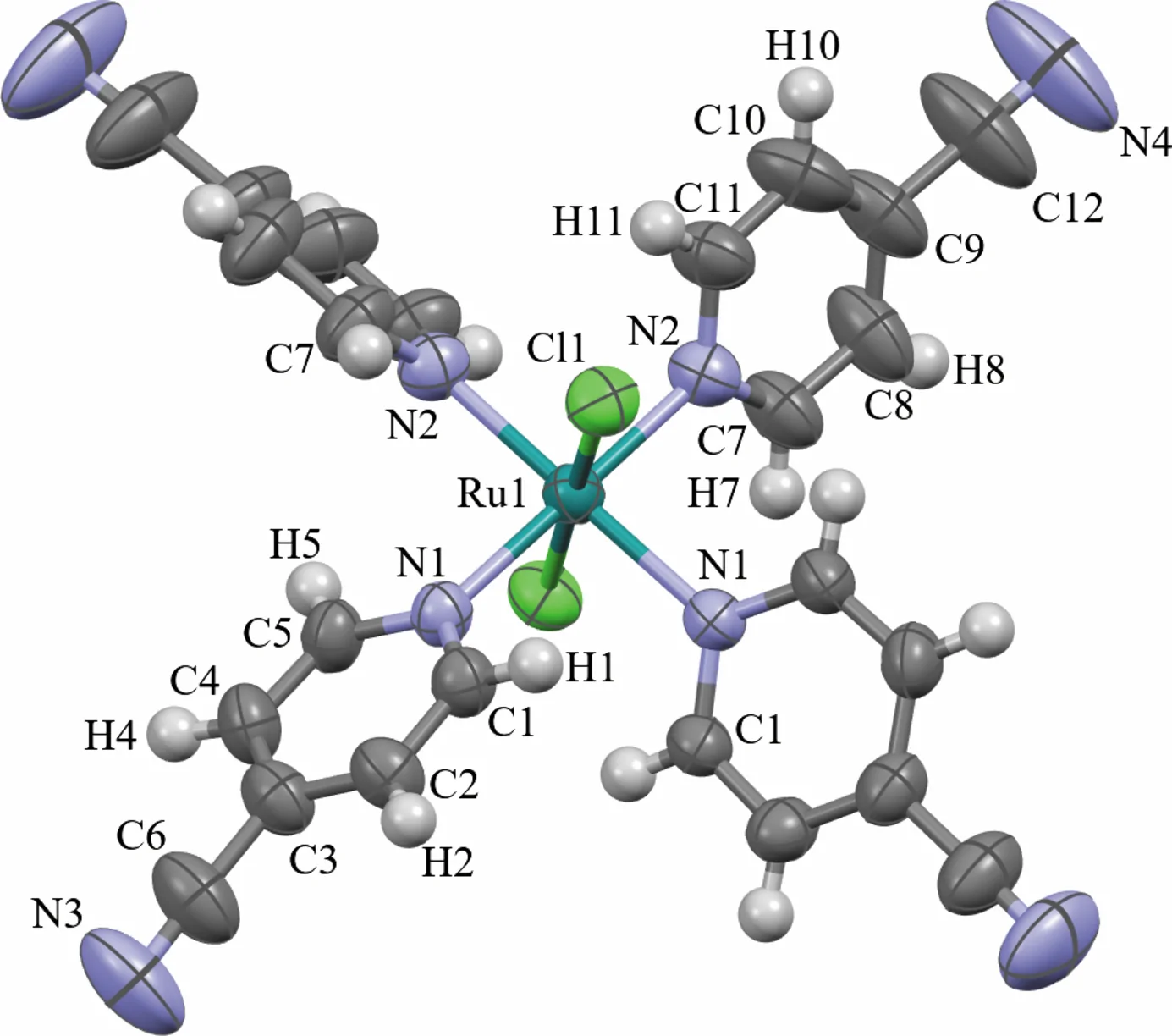
The propeller-like arrangement of the two unique pcp ligands in *trans*-[Ru(pcp)_4_Cl_2_] (ellipsoids at 50% probability) along with labels of the symmetry-generated (1 − *x*, *y*, 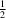 − *x*) N1, C1 N2 and C7 atoms.>

The ruthenium atom, and thereby the title complex, lies on a twofold axis of the *C*2/*c* space group. The structure of the complex has the four pcp ligands in a propeller-like arrangement around the ruthenium atom (Fig. 1 ▸), which is typical of ruthenium complexes with four pyridyl-based ligands (Małecki *et al.*, 2005 ▸; Wong & Lau, 1994 ▸). The tilt angle of the pyridyl rings compared to the plane containing Ru and the four coordinated N atoms is 40.78 (11)° (N1-pyridyl ring) and 55.47 (12)° (N2- pyridyl ring). The ruthenium–pyridyl nitro­gen distances are 2.085 (3) and 2.082 (3) Å, which fall in the range of Ru—N distances in other ruthenium(II) complexes with four pyridyl-based ligands: for example, 2.0620 (14) Å for pyrazine (Nesterov *et al.*, 2012 ▸), 2.080 (3) Å for pyridine (Coe *et al.*, 1995 ▸), and 2.137 (5) Å for 4-meth­oxy­pyridine (Reinheimer *et al.*, 2023 ▸). The Ru—N distance is shorter compared to other ruthenium(II) complexes containing one pcp ligand, many of which utilize larger *trans*-influence ligands like η^6^-arenes, such as [Ru(η^6^-C_6_Me_6_)Cl_2_(pcp)], 2.136 (6) Å (Singh *et al.*, 2007 ▸) or [Ru(η^6^-(*p*-cymene))I_2_(pcp)], 2.119 (6) Å (Torubaev & Skabitsky, 2019 ▸), or an η^3^-(all­yl) ligand, for example in [Ru(η^3^:η^3^-C_10_H_16_)Cl_2_(pcp)], 2.210 (3) and 2.202 (3) Å (Nand Sahay *et al.*, 2000 ▸).

**Figure 2:**
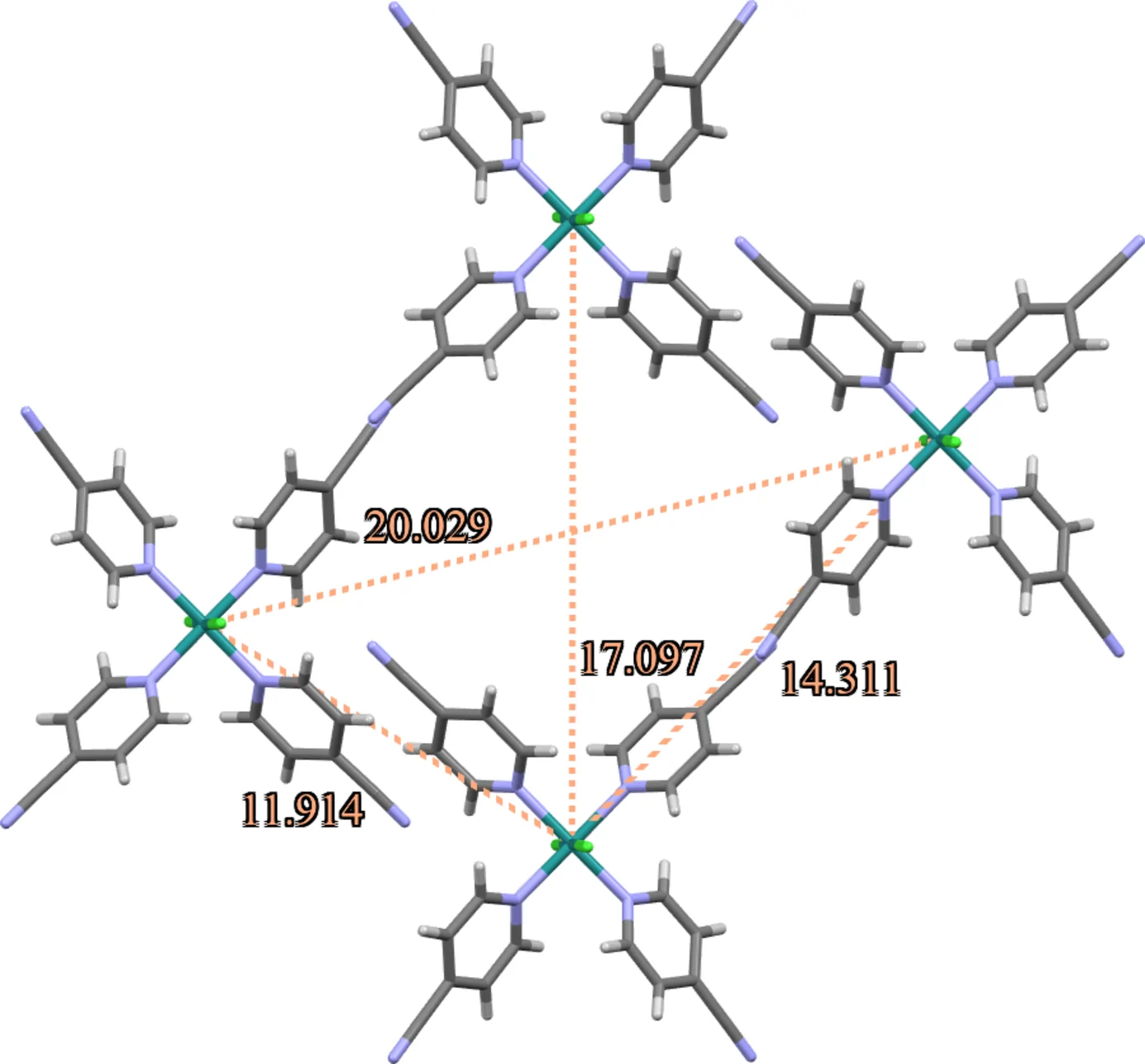
Representative packing of *trans*-[Ru(pcp)_4_Cl_2_] (capped sticks) viewed along the *c* axis of the grid-like assembly with inter­molecular Ru⋯Ru distances (Å) within a unit of the grid.

**Figure 3:**
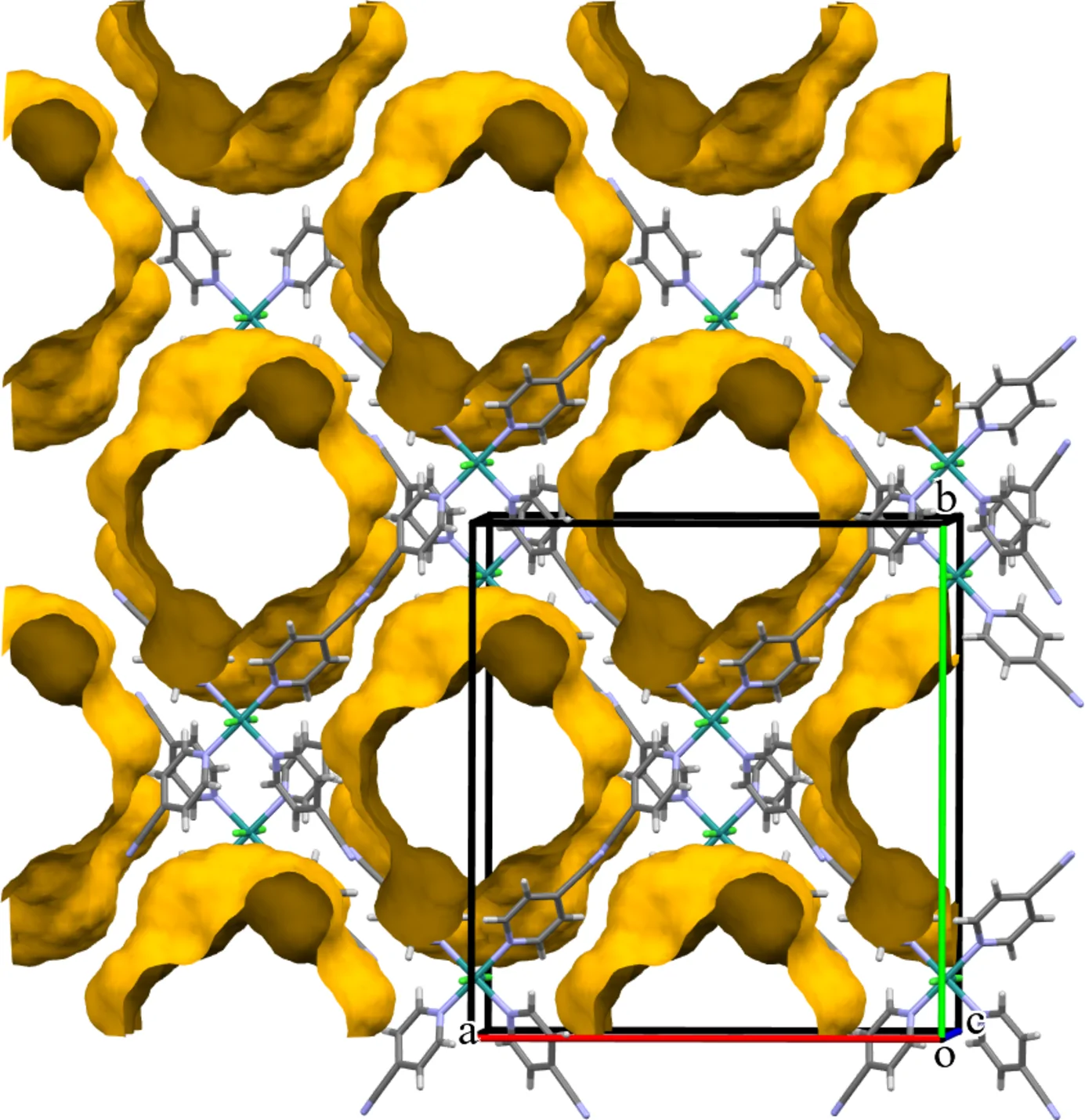
Calculated voids (yellow surface) in crystal unit cell (axes labelled) with capped stick model.

**Figure 4:**
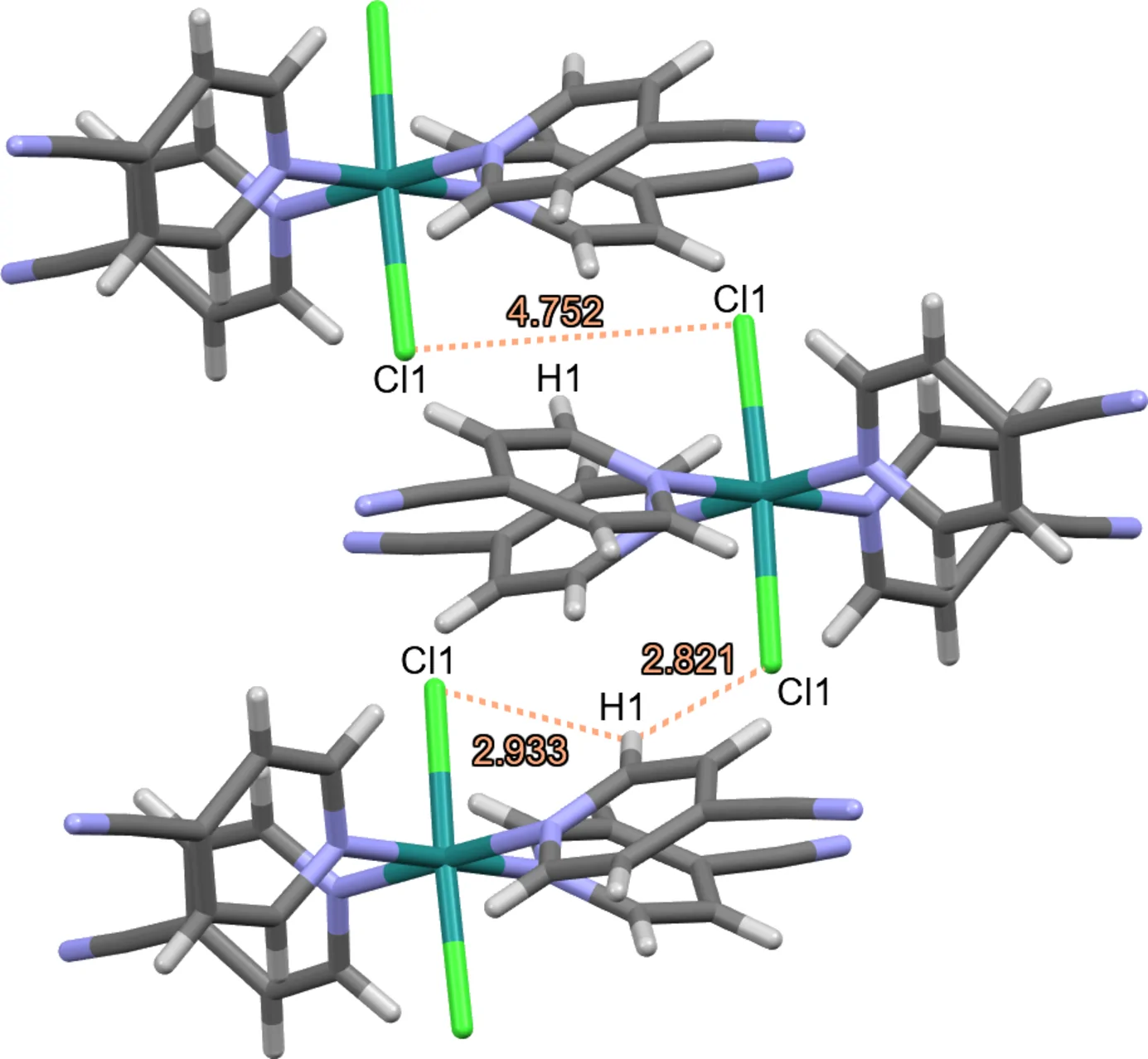
The staggered stacking of the complexes (capped sticks) showing the longer Cl1⋯H1 intra­molecular distance (2.93 Å) and the inter­molecular distance of the shorter Cl1(1 − *x*, 1 − *y*, 1 − *z*)⋯H1 (2.82 Å) and Cl1(1 − *x*, *y*, 1.5 − *z*)⋯Cl1(*x*, 1 − *y*, 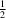 + *z*) (4.752 Å).

The title complex packs in a grid-like assembly resulting from the overlap of the carbonitrile groups of ruthenium-pcp units. The Ru⋯Ru′ distances within a grid are 11.9141 (5) (Ru′ = 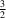 − *x*, 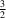 − *y*, 1 − *z*) and 14.3106 (3) Å (Ru′ = −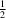 + *x*, 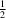 + *y*, *z*) and Ru′⋯Ru′′ diagonal distances of 17.0973 (9) (Ru′ = *x*, *y*, *z* and Ru′′ = 1 − *x*, 2 − *y*, 1 − *z*) and 20.0289 (5) Å (Ru′ = −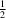 + *x*, 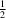 + *y*, *z* and Ru′′ = 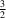 − *x*, 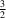 − *y*, 1 − *z*) (Fig. 2 ▸). The calculated void volume (Macrae *et al.*, 2020 ▸) is 47.0% (1924.60 Å^3^) of the unit cell (Fig. 3 ▸), which is presumably occupied by disordered solvent molecules. The complexes stack in a staggered pattern along the direction of the Cl—Ru—Cl axes with inter­molecular distance between the two chloride ions [Cl1 at *x*, *y*, *z* and symmetry-related Cl1 at 1 − *x*, 1 − *y*, 1 − *z*] of 4.7515 (17) Å. The chloride ions of one complex are near the H1 atoms of neighbouring complexes with the distance between Cl1(1 − *x*, 1 − *y*, 1 − *z*) and H1 being closer to the neighbouring H1 atom (2.82 Å) than the corresponding intra­molecular H1 atom (2.93 Å) (Fig. 4 ▸).

## Synthesis and crystallization

Following the preparation of other *trans*-RuCl_2_ complexes containing four pyridyl ligands, (Coe *et al.*, 1995 ▸; Coe, 2004 ▸) the title compound was synthesized by mixing, at reflux, a yellow solution containing 100 mg (0.206 mmol) of [Ru(DMSO)_4_Cl_2_], and 99.6 mg (0.957 mmol) of pyridine-4-carbo­nitrile in 20 ml of toluene for 2 h under N_2_ with light excluded. The resulting dark-red solution slowly cooled and sat undisturbed for one week. The resulting dark red–brown solid was filtered in air and washed with toluene to give 90 mg (74% yield) of product. FT–IR 2921(C—H), 2234(C≡N), 1606, 1485, 1420, 1307, 1219, 1110, 1095, 1017, 936, 833, 732, 716, 683, 564, 426 cm^−1. 1^H-NMR (acetone-*d*_6_, δ/p.p.m.) 8.69 (*dd*), 7.62 (*dd*), 7.2–7.0 (toluene), 2.28 (toluene). Dark-red block-shaped crystals were harvested directly from the product of *trans*-[Ru(pcp)_4_Cl_2_] and used for X-ray structural characterization, as well as for FT–IR and NMR spectroscopy analyses.

## Refinement

Crystal data, data collection and structure refinement details are summarized in Table 1 ▸. A solvent mask was calculated and 92 electrons were found in the void per unit cell (Dolomanov *et al.*, 2009 ▸). This is consistent with the presence of two toluene mol­ecules per unit cell, which accounts for 100 electrons per unit cell. In the ^1^H-NMR using crystals (noted in the *Synthesis and crystallization* section), we observed an integration of 2 toluene molecules per [Ru(pcp)_4_Cl_2_], but the electron density over the duration of the 293  K data collection of a single crystal resulted in a toluene hemisolvate.

## Supplementary Material

Crystal structure: contains datablock(s) I. DOI: 10.1107/S2414314626009375/bh4104sup1.cif

Structure factors: contains datablock(s) I. DOI: 10.1107/S2414314626009375/bh4104Isup2.hkl

CCDC reference: 2586520

Additional supporting information:  crystallographic information; 3D view; checkCIF report

## Figures and Tables

**Table 1:** Experimental details

Crystal data	
Chemical formula	[RuCl_2_(C_6_H_4_N_2_)_4_]·0.5C_7_H_8_
*M*_r_	588.42
Crystal system, space group	Monoclinic, *C*2/*c*
Temperature (K)	293
*a*, *b*, *c* (Å)	19.3087 (5), 21.1269 (6), 10.0865 (2)
β (°)	95.909 (2)
*V* (Å^3^)	4092.75 (18)
*Z*	4
Radiation type	Mo *K*α
μ (mm^−1^)	0.54
Crystal size (mm)	0.25 × 0.23 × 0.18

Data collection	
Diffractometer	XtaLAB Mini II
Absorption correction	Analytical (*CrysAlis PRO*; Rigaku OD, 2022 ▸)
*T*_min_, *T*_max_	0.899, 0.929
No. of measured, independent and observed [*I* > 2σ(*I*)] reflections	43912, 3647, 3113
*R*_int_	0.037
(sin θ/λ)_max_ (Å^−1^)	0.598

Refinement	
*R*[*F*^2^ > 2σ(*F*^2^)], *wR*(*F*^2^), *S*	0.048, 0.146, 1.01
No. of reflections	3647
No. of parameters	159
H-atom treatment	H-atom parameters constrained
Δρ_max_, Δρ_min_ (e Å^−3^)	0.82, −0.58

Computer programs: *CrysAlis PRO* (Rigaku OD, 2022 ▸), *SHELXT* (Sheldrick, 2015*a* ▸), *SHELXL2014/7* (Sheldrick, 2015*b* ▸), *OLEX2* (Dolomanov *et al.*, 2009 ▸) and *Mercury* (Macrae *et al.*, 2020 ▸).

## full crystallographic data

### trans-Dichloridotetrakis(pyridine-4-carbonitrile-κN)ruthenium(II) toluene hemisolvate Crystal data

**Table:**

[RuCl_2_(C_6_H_4_N_2_)_4_]·0.5C_7_H_8_	*F*(000) = 1276
*M**_r_* = 588.42	*D*_x_ = 0.955 Mg m^−^^3^
Monoclinic, *C*2/*c*	Mo *K*α radiation, λ = 0.71073 Å
*a* = 19.3087 (5) Å	Cell parameters from 7651 reflections
*b* = 21.1269 (6) Å	θ = 2.0–22.1°
*c* = 10.0865 (2) Å	µ = 0.54 mm^−^^1^
β = 95.909 (2)°	*T* = 293 K
*V* = 4092.75 (18) Å^3^	Block, red
*Z* = 4	0.25 × 0.23 × 0.18 mm

### trans-Dichloridotetrakis(pyridine-4-carbonitrile-κN)ruthenium(II) toluene hemisolvate Data collection

**Table:**

XtaLAB Mini II diffractometer	3647 independent reflections
Radiation source: fine-focus sealed X-ray tube, Rigaku (Mo) X-ray Source	3113 reflections with *I* > 2σ(*I*)
Graphite monochromator	*R*_int_ = 0.037
Detector resolution: 10.0000 pixels mm^-1^	θ_max_ = 25.2°, θ_min_ = 2.4°
ω scans	*h* = −23→23
Absorption correction: analytical (CrysAlisPro; Rigaku OD, 2022)	*k* = −25→25
*T*_min_ = 0.899, *T*_max_ = 0.929	*l* = −12→12
43912 measured reflections	

### trans-Dichloridotetrakis(pyridine-4-carbonitrile-κN)ruthenium(II) toluene hemisolvate Refinement

**Table:**

Refinement on *F*^2^	Primary atom site location: dual
Least-squares matrix: full	Secondary atom site location: difference Fourier map
*R*[*F*^2^ > 2σ(*F*^2^)] = 0.048	Hydrogen site location: inferred from neighbouring sites
*wR*(*F*^2^) = 0.146	H-atom parameters constrained
*S* = 1.01	*w* = 1/[σ^2^(*F*_o_^2^) + (0.1014*P*)^2^ + 3.5296*P*] where *P* = (*F*_o_^2^ + 2*F*_c_^2^)/3
3647 reflections	(Δ/σ)_max_ < 0.001
159 parameters	Δρ_max_ = 0.82 e Å^−^^3^
0 restraints	Δρ_min_ = −0.58 e Å^−^^3^
0 constraints	

### trans-Dichloridotetrakis(pyridine-4-carbonitrile-κN)ruthenium(II) toluene hemisolvate Fractional atomic coordinates and isotropic or equivalent isotropic displacement parameters (Å^2^)

**Table:**

	*x*	*y*	*z*	*U*_iso_*/*U*_eq_	
Ru1	0.5000	0.61337 (2)	0.2500	0.04687 (18)	
Cl1	0.47985 (5)	0.61078 (4)	0.48123 (9)	0.0612 (3)	
N1	0.42424 (14)	0.54388 (13)	0.2100 (3)	0.0490 (6)	
N2	0.57540 (17)	0.68344 (14)	0.2886 (3)	0.0584 (7)	
N3	0.2566 (3)	0.3494 (3)	0.0460 (5)	0.130 (2)	
N4	0.7718 (4)	0.8570 (4)	0.4127 (7)	0.184 (4)	
C1	0.42393 (18)	0.48927 (17)	0.2810 (3)	0.0532 (8)	
H1	0.4545	0.4858	0.3581	0.064*	
C2	0.3816 (2)	0.43937 (19)	0.2459 (4)	0.0606 (9)	
H2	0.3848	0.4024	0.2961	0.073*	
C3	0.3337 (2)	0.4443 (2)	0.1343 (4)	0.0655 (10)	
C4	0.3301 (2)	0.5013 (2)	0.0642 (4)	0.0645 (10)	
H4	0.2968	0.5071	−0.0083	0.077*	
C5	0.37586 (18)	0.54852 (18)	0.1033 (4)	0.0564 (9)	
H5	0.3738	0.5858	0.0540	0.068*	
C6	0.2899 (3)	0.3921 (3)	0.0871 (5)	0.0898 (16)	
C7	0.6328 (2)	0.6852 (2)	0.2254 (4)	0.0753 (12)	
H7	0.6386	0.6543	0.1617	0.090*	
C8	0.6837 (3)	0.7301 (3)	0.2495 (5)	0.0941 (16)	
H8	0.7223	0.7308	0.2014	0.113*	
C9	0.6757 (3)	0.7745 (2)	0.3482 (5)	0.0936 (16)	
C10	0.6169 (3)	0.7736 (2)	0.4120 (5)	0.0973 (17)	
H10	0.6105	0.8037	0.4769	0.117*	
C11	0.5674 (2)	0.7283 (2)	0.3804 (5)	0.0759 (12)	
H11	0.5271	0.7285	0.4235	0.091*	
C12	0.7309 (4)	0.8217 (4)	0.3820 (6)	0.138 (3)	

### trans-Dichloridotetrakis(pyridine-4-carbonitrile-κN)ruthenium(II) toluene hemisolvate Atomic displacement parameters (Å^2^)

**Table:**

	*U*^11^	*U*^22^	*U*^33^	*U*^12^	*U*^13^	*U*^23^
Ru1	0.0492 (3)	0.0439 (3)	0.0466 (3)	0.000	0.00040 (16)	0.000
Cl1	0.0703 (6)	0.0627 (6)	0.0507 (5)	0.0067 (4)	0.0063 (4)	0.0019 (4)
N1	0.0483 (15)	0.0487 (16)	0.0492 (15)	0.0007 (12)	0.0012 (12)	0.0047 (12)
N2	0.0640 (19)	0.0546 (17)	0.0567 (18)	−0.0045 (14)	0.0060 (15)	−0.0052 (14)
N3	0.142 (5)	0.131 (4)	0.111 (4)	−0.073 (4)	−0.008 (3)	−0.014 (3)
N4	0.217 (7)	0.206 (7)	0.126 (5)	−0.152 (7)	0.003 (5)	−0.026 (5)
C1	0.0546 (19)	0.055 (2)	0.0490 (19)	−0.0004 (16)	0.0022 (15)	0.0065 (16)
C2	0.068 (2)	0.060 (2)	0.052 (2)	−0.0116 (18)	0.0035 (17)	0.0045 (17)
C3	0.063 (2)	0.071 (3)	0.063 (2)	−0.016 (2)	0.0067 (18)	−0.0044 (19)
C4	0.053 (2)	0.077 (3)	0.061 (2)	−0.0067 (19)	−0.0062 (17)	0.002 (2)
C5	0.0504 (19)	0.059 (2)	0.059 (2)	0.0030 (16)	−0.0011 (16)	0.0092 (17)
C6	0.095 (4)	0.100 (4)	0.073 (3)	−0.040 (3)	0.004 (3)	−0.002 (3)
C7	0.078 (3)	0.076 (3)	0.072 (3)	−0.028 (2)	0.006 (2)	−0.008 (2)
C8	0.092 (3)	0.112 (4)	0.078 (3)	−0.051 (3)	0.006 (3)	−0.005 (3)
C9	0.116 (4)	0.084 (3)	0.079 (3)	−0.052 (3)	0.000 (3)	−0.002 (3)
C10	0.139 (5)	0.066 (3)	0.088 (3)	−0.033 (3)	0.013 (3)	−0.014 (2)
C11	0.085 (3)	0.061 (2)	0.082 (3)	−0.014 (2)	0.012 (2)	−0.011 (2)
C12	0.174 (6)	0.149 (6)	0.088 (4)	−0.107 (5)	−0.001 (4)	−0.007 (4)

### trans-Dichloridotetrakis(pyridine-4-carbonitrile-κN)ruthenium(II) toluene hemisolvate Geometric parameters (Å, º)

**Table:**

Ru1—Cl1^i^	2.4044 (9)	C2—C3	1.386 (5)
Ru1—Cl1	2.4044 (9)	C3—C4	1.395 (6)
Ru1—N1	2.082 (3)	C3—C6	1.441 (6)
Ru1—N1^i^	2.082 (3)	C4—H4	0.9300
Ru1—N2	2.085 (3)	C4—C5	1.364 (5)
Ru1—N2^i^	2.085 (3)	C5—H5	0.9300
N1—C1	1.358 (4)	C7—H7	0.9300
N1—C5	1.355 (4)	C7—C8	1.368 (6)
N2—C7	1.335 (5)	C8—H8	0.9300
N2—C11	1.345 (5)	C8—C9	1.389 (7)
N3—C6	1.159 (6)	C9—C10	1.361 (7)
N4—C12	1.108 (7)	C9—C12	1.474 (7)
C1—H1	0.9300	C10—H10	0.9300
C1—C2	1.358 (5)	C10—C11	1.368 (6)
C2—H2	0.9300	C11—H11	0.9300
			
Cl1—Ru1—Cl1^i^	177.39 (5)	C2—C3—C4	118.1 (4)
N1—Ru1—Cl1	89.49 (8)	C2—C3—C6	122.1 (4)
N1—Ru1—Cl1^i^	88.67 (8)	C4—C3—C6	119.8 (4)
N1^i^—Ru1—Cl1^i^	89.49 (8)	C3—C4—H4	120.4
N1^i^—Ru1—Cl1	88.67 (8)	C5—C4—C3	119.2 (3)
N1—Ru1—N1^i^	90.34 (15)	C5—C4—H4	120.4
N1^i^—Ru1—N2^i^	179.48 (11)	N1—C5—C4	123.4 (3)
N1—Ru1—N2	179.48 (11)	N1—C5—H5	118.3
N1—Ru1—N2^i^	90.07 (12)	C4—C5—H5	118.3
N1^i^—Ru1—N2	90.07 (12)	N3—C6—C3	177.4 (7)
N2—Ru1—Cl1^i^	91.01 (9)	N2—C7—H7	118.3
N2^i^—Ru1—Cl1^i^	90.84 (9)	N2—C7—C8	123.4 (4)
N2^i^—Ru1—Cl1	91.02 (9)	C8—C7—H7	118.3
N2—Ru1—Cl1	90.84 (9)	C7—C8—H8	121.2
N2^i^—Ru1—N2	89.51 (17)	C7—C8—C9	117.7 (5)
C1—N1—Ru1	122.6 (2)	C9—C8—H8	121.2
C5—N1—Ru1	121.1 (2)	C8—C9—C12	119.4 (6)
C5—N1—C1	116.1 (3)	C10—C9—C8	119.3 (4)
C7—N2—Ru1	121.9 (3)	C10—C9—C12	121.2 (5)
C7—N2—C11	118.0 (4)	C9—C10—H10	120.1
C11—N2—Ru1	120.1 (3)	C11—C10—C9	119.7 (5)
N1—C1—H1	118.0	C11—C10—H10	120.1
C2—C1—N1	123.9 (3)	N2—C11—C10	121.8 (5)
C2—C1—H1	118.0	N2—C11—H11	119.1
C1—C2—H2	120.4	C10—C11—H11	119.1
C1—C2—C3	119.1 (4)	N4—C12—C9	177.1 (7)
C3—C2—H2	120.4		
			
Ru1—N1—C1—C2	−170.2 (3)	C3—C4—C5—N1	−1.9 (6)
Ru1—N1—C5—C4	172.5 (3)	C5—N1—C1—C2	4.1 (5)
Ru1—N2—C7—C8	−179.2 (4)	C6—C3—C4—C5	−174.4 (4)
Ru1—N2—C11—C10	177.5 (4)	C7—N2—C11—C10	−1.8 (6)
N1—C1—C2—C3	−2.6 (6)	C7—C8—C9—C10	−3.0 (8)
N2—C7—C8—C9	2.2 (8)	C7—C8—C9—C12	176.9 (5)
C1—N1—C5—C4	−1.8 (5)	C8—C9—C10—C11	1.4 (9)
C1—C2—C3—C4	−1.3 (6)	C9—C10—C11—N2	1.0 (8)
C1—C2—C3—C6	176.5 (4)	C11—N2—C7—C8	0.1 (7)
C2—C3—C4—C5	3.4 (6)	C12—C9—C10—C11	−178.5 (5)

Symmetry code: (i) −*x*+1, *y*, −*z*+1/2.
